# A review of the impact of obesity on common gastrointestinal malignancies

**DOI:** 10.15761/ICST.1000223

**Published:** 2017-01-18

**Authors:** Somashekar G. Krishna, Hisham Hussan, Zobeida Cruz-Monserrate, Lanla F. Conteh, Khalid Mumtaz, Darwin L. Conwell

**Affiliations:** 1 Sections of Pancreatic Disorders and Advanced Endoscopy, Division of Gastroenterology, Hepatology and Nutrition, Comprehensive Cancer Center, The Ohio State University Wexner Medical Center, USA; 2 Sections of Intestinal Neoplasia and Hereditary Polyposis, and Obesity and Bariatric Endoscopy, Division of Gastroenterology, Hepatology and Nutrition, Comprehensive Cancer Center, The Ohio State University, USA; 3 Section of Molecular and Cellular Biology, Division of Gastroenterology, Hepatology and Nutrition, Comprehensive Cancer Center, The Ohio State University Wexner Medical Center, USA; 4 Section of Hepatology & Comprehensive Transplant Center, Division of Gastroenterology, Hepatology and Nutrition, Comprehensive Cancer Center, The Ohio State University Wexner Medical Center, USA; 5 Section of Pancreatic Disorders, Division of Gastroenterology, Hepatology and Nutrition, Comprehensive Cancer Center, The Ohio State University Wexner Medical Center, USA

## Abstract

Obesity is a global pandemic and is a well-recognized risk factor for various gastrointestinal diseases. The prevalence of obesity is increasing across all age groups. There is an emergent need for focused guidelines aimed at reducing the incidence, prevalence, and associated risks of obesity. The impact of obesity on gastrointestinal cancers being multifactorial adversely influences the associated risk, disease course, prognosis, and overall survival. We have summarized the current literature highlighting the association between obesity and common gastrointestinal cancers, with specific focus on esophageal adenocarcinoma, colon cancer, hepatocellular cancer, cholangiocarcinoma, and pancreatic malignancies.

## Introduction

Nearly 30% of the world’s population is overweight or obese, and none of the countries in the world have been successful in reducing the obesity rates over the last 33 years [1]. In the United States (U.S), obesity has doubled in the last two decades and amounts to 78.6 million (34.9%) of adults [2]. The prevalence of morbid obesity (Body Metabolic Index [BMI] > 40) is rapidly increasing with an approximate 70% growth in the last decade [3]. Likewise, childhood obesity which predicts the impending future, has more than doubled in children and quadrupled in adolescents in the last three decades [2].

The evidence linking the obesity pandemic to various gastrointestinal cancers is mounting. The data are a mixture of observational, retrospective and a few prospective studies. A forecast of the imminent health and economic burden of obesity in 2030 estimated that continuation of prevailing accumulative trends in obesity will lead to about 500,000 additional cases of cancer in the U.S. by 2030 [4]. Obesity not only increases cancer risk, but also diminishes survival of cancer patients [5]. Although the relationship is not linear for a few malignancies, continued research is warranted to establish clear risks and associations. This review summarizes the current literature associating obesity to gastrointestinal cancers, specifically, cholangiocarcinoma, esophageal, gastric, hepatocellular, pancreatic, and colorectal malignancies.

## Esophageal cancer

Several studies have reported that overweight and obese patients are more often diagnosed with esophageal adenocarcinoma. A Mendelian randomized study analyzing 999 patients with esophageal adenocarcinoma, 2,061 patients with Barrett’s esophagus, and 2,169 population controls demonstrated that the risk of adenocarcinoma of the esophagus and Barrett’s esophagus increased by 16% and 12% per 1 kg/m^2^ increase in BMI, respectively [6]. A meta-analysis has demonstrated that a higher BMI range (BMI of 30 to 34.9 kg/m^2^ vs. <25 kg/m^2^) contributed to a 2.4-fold increase in the risk of esophageal adenocarcinoma [7]. The mechanisms of above associations are multifold – obesity increases intraabdominal pressure and risk of hiatal hernia and esophageal reflux disease, there are increased circulating levels of cytokines in patients with central obesity, associated metabolic syndrome is associated with Barrett’s esophagus and adenocarcinoma, and a complex milieu of metabolic disorders in obesity appears to propel esophageal reflux disease to Barrett’s esophagus and subsequent adenocarcinoma [8–12]. In addition to being a risk factor, there is accumulating evidence that obesity at the time of diagnosis of esophageal adenocarcinoma is an adverse prognostic characteristic influencing treatment outcomes [13].

## Colorectal cancer

Colorectal cancer (CRC) is the third leading cause of cancer incidence and mortality in the U.S [14]. Obesity is one of the strongest risk factors for colorectal neoplasia development accompanied by incremental risk with every 5-unit increase in BMI [15–19]. Experimental and epidemiological studies identified obesity as an independent risk factor for CRC irrespective of the impact of diet and exercise [20,21]. Furthermore, obesity’s impact on CRC development was higher in men compared to women and more commonly seen in the colon compared to rectum [21,22]. This could be due to the differential impact of obesity in relation to colorectal embryonic origins or sex hormone levels. This well-established causality between obesity and CRC is of particular interest with the rising rates of obesity that can explain the incremental CRC incidence in patients younger than 50 of age in the U.S [23,24].

The underlying mechanisms linking obesity to CRC are likely multifaceted. One well defined pathway is increased insulin resistance that has been associated with colorectal adenomas, CRC progression as well as larger tumor size [25–29]. This link was hypothesized to be through increased insulin-like growth factor 1 (IGF-I) levels and bioavailability seen with high insulin resistance [28,30]. Another major mechanism responsible for CRC is low grade inflammation seen in obesity with increased IL-1b, IL-6, TNF-a and NF-kB [31–33]. Finally, obesity is associated with leptin and decreased adiponectin [34]. Leptin can signal through the carcinogenic (JAK/STAT) pathway while adiponectin reduces pro-inflammatory cytokines via inhibition of (NF-kB) [35]. Indeed, a 3-fold increase in colon cancer risk was seen with increasing concentrations of leptin [36]. In addition to increase CRC incidence, obesity was associated with worse CRC outcomes. For instance, there was an increase in CRC-surgery perioperative morality, surgical complications and health-care utilization in association with morbid obesity [37]. Additionally, obesity was found to be associated with a greater risk of CRC recurrence following CRC diagnosis and treatment [38,39]. Finally, obesity was associated with inferior long term overall survival [40,41].

## Pancreatic cancer

Obesity is a significant risk factor for pancreatic adenocarcinoma (PDAC) [42–46]. This is concerning, as obesity rates are increasing worldwide, mostly due to increased consumption of a Western-style diet, high in fat and calories [47]. Epidemiological data indicates that preexisting obesity adversely influences PDAC–related mortality in a dose-dependent manner [5]. A meta-analysis and a pooled analysis of PDAC patients demonstrated a 10% and 14% increase in risk for each 5 kg/m^2^ (above baseline of 30 kg/m^2^) incremental increase in BMI, respectively [48,49]. A more recent study observed positive associations among measures of central obesity, waist circumference, and waist-to-hip ratio with pancreatic cancer mortality [50].

Although many epidemiological studies have revealed association of obesity with PDAC affecting surgical outcomes, many issues about the underlying mechanisms remain unanswered. Utilizing genetically engineered mouse models, it has been demonstrated that diet-induced obesity acts as an inflammatory stimulus to trigger increased *KRas* activity [51]. Cyclooxygenase-2 (Cox-2) was also found to be critical in the inflammatory loop that leads to inflammation, increased fibrotic stroma, activation of *KRas* downstream signaling pathways, increases development of pancreatic intraepithelial neoplasia (PanIN) lesions, and decrease mice survival [51]. In addition, recent studies have demonstrated that increasing visceral obesity and associated sarcopenia adversely influence postoperative morbidity and long-term survival in PDAC [44,52–54]. Future research looking at factors derived from adipose tissue that could be promoting the development of PDAC are critical in an effort to develop preventative strategies.

## Hepatocellular cancer

The incidence of hepatocellular carcinoma (HCC) is rapidly increasing and it is now the second most common cause of cancer related deaths worldwide. In 2016, it is estimated that more than 35,000 people in the U.S alone will be diagnosed with HCC. This number has been steadily increasing by about 3–4% annually over the last 10 years; overall, the incidence has tripled since the 1980s [55,56]. Viral hepatitis was once the leading cause of HCC worldwide. However, non-alcoholic fatty liver disease (NAFLD) has now been recognized as a leading agent in the HCC epidemic. NAFLD affects more than 80 million Americans and ranges from simple hepatic steatosis to nonalcoholic steatohepatitis (NASH) which involves inflammation and injury at the cellular level and can progress to fibrosis and eventually cirrhosis. The rising prevalence of NAFLD directly reflects that of the metabolic syndrome and obesity pandemics [57]. Available data suggests that there is a 1.5 to 4 fold increased risk of HCC with obesity [58]. The mechanisms by which carcinogenesis is promoted remain unclear, however, proinflammatory cytokines, adinopectin, and insulin resistance have all identified as potential role players [57,59].

Diabetes mellitus alone has been identified as a negative prognostic indicator in patients with HCC. These patients have been shown to have a higher incidence of distant metastatic disease and a higher incidence of histological macrovascular invasion [60]. Although more prevalent in patients with cirrhosis, it is important to recognize that HCC can develop in patients with NAFLD in the absence of cirrhosis. Furthermore, there is increasing evidence that not only does NAFLD increase the risk of HCC, it may also increase the risk for tumor recurrence after treatment with locoregional therapy when compared to other etiologies of cirrhosis and HCC [61].

## Cholangiocarcinoma

Data on association and pathogenesis of obesity with cholangiocarcinoma (CC) is emerging and, few epidemiologic studies have tried to assess this link [62–64]. Analysis of the Surveillance, Epidemiology, and End Results (SEER) – Medicare database reported a significant association between obesity and intrahepatic CC, but not between obesity and extrahepatic CC [63]. However, the significant limitation of this study was inclusion of patients ≥ 65 years which might not be generalized to younger population. Also large database studies have chances of diagnostic bias due to the fact that obese people with various diseases are more likely to undergo testing and thus higher likelihood of a specific diagnosis. A Danish population-based study did not find significant association between obesity and CC [64]. This study has strengths of using complete national registry and histological confirmation of CC diagnosis. A study of the primary care database from the United Kingdom reported that patients with BMI ≥ 30 kg/m^2^ had 1.5 times the risk of CC compared with those with BMI <25 kg/m^2^ [62]. More recently, a meta-analysis (five cohort and five case-control studies) revealed that being overweight (pooled OR 1.30, 95 % CI 1.13–1.49) and obese (pooled OR 1.52, 95 % CI 1.13–1.89) were significantly associated with CC [65].

Major physiological site of leptin action is in central nervous system. However, the receptors are also expressed in peripheral tissues including cholangiocytes [66]. Studies have demonstrated that serum leptin levels are increased in obesity and have been suggested as a risk factor for CC [66]. Besides leptin, other pro-inflammatory cytokines - Interleukin-6 (IL-6) and tumor necrosis factor (TNF) are linked strongly to obesity and have been found to have a role in development of CC [56]. Based on the current literature review, evidence of association of obesity with CC is growing and needs to be confirmed with long-term cohort studies. Research on the causal role of obesity and progression of different cancers including cholangiocarcinoma is evolving.

## Conclusion

Obesity impacts the risk and outcomes of a broad spectrum of gastrointestinal diseases including esophageal reflux disease, nonalcoholic fatty liver disease, gallstone disease, and acute pancreatitis. As detailed in this review, this global pandemic also adversely influences the risk and prognosis of related gastrointestinal pre-malignant and malignant conditions. The influence of obesity appears to be a mixture of mechanical, humoral, pro-inflammatory, and complex metabolic mechanisms. These associations are worrisome since recent trends indicate increasing prevalence of obesity in children and adolescents. The implications include gastrointestinal malignancies which are difficult to manage especially in a younger patient population. Hence, there is an emergent need for focused research on increasing and improving operative, endoscopic, and non-invasive interventions for effectively containing the uncontrolled global surge in prevalence of obesity.
